# Supplementary material for: A rapid, cost-effective tailed amplicon method for sequencing SARS-CoV-2
Source: BMC Genomics. 2020 Dec 4;21:863. doi: 10.1186/s12864-020-07283-6 (PMC7716288; doi:10.1186/s12864-020-07283-6)
Supplement: Supplementary file 1 [file 12864_2020_7283_MOESM1_ESM.docx]

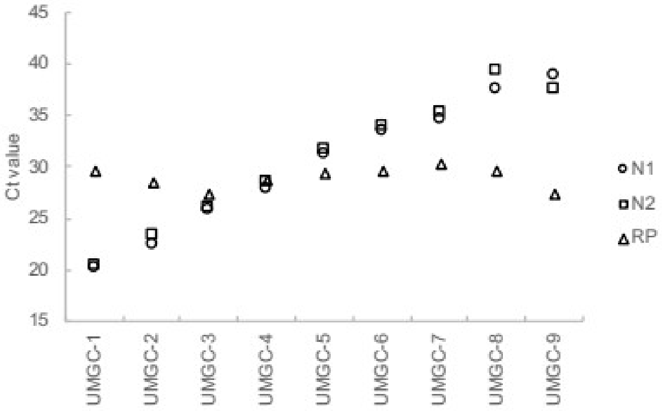

Supplemental Figure S1

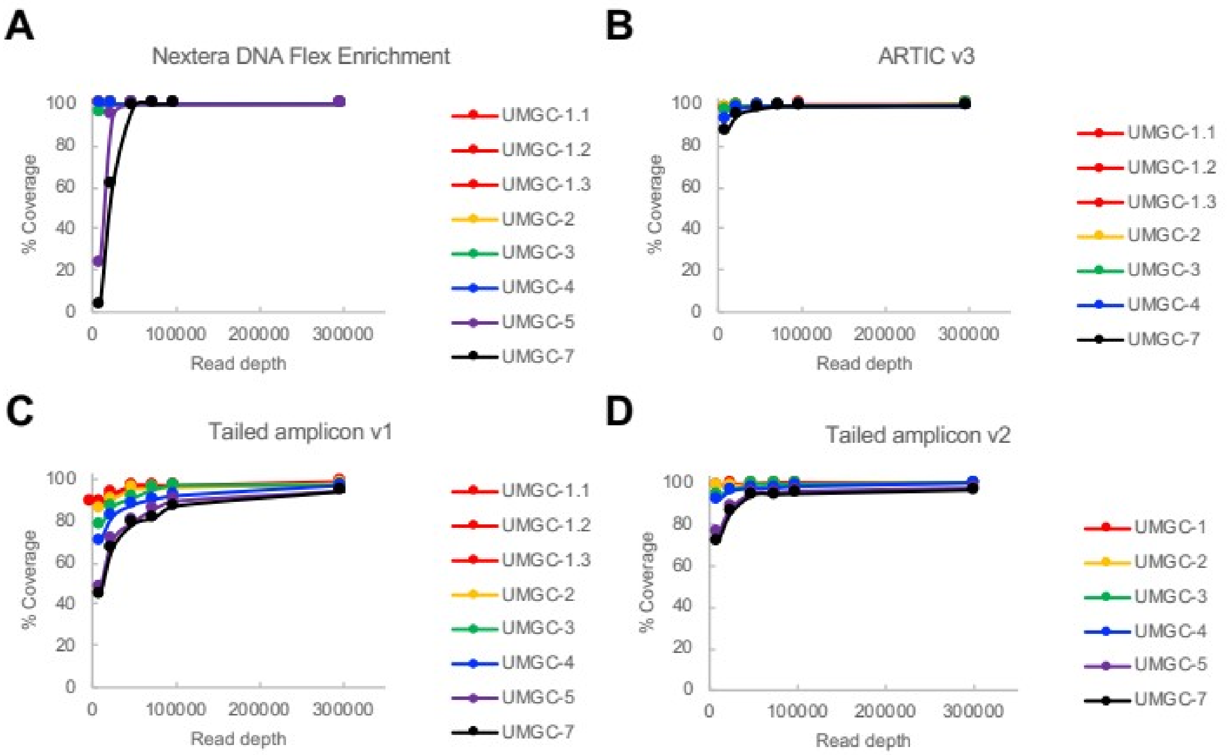

Supplemental Figure S2

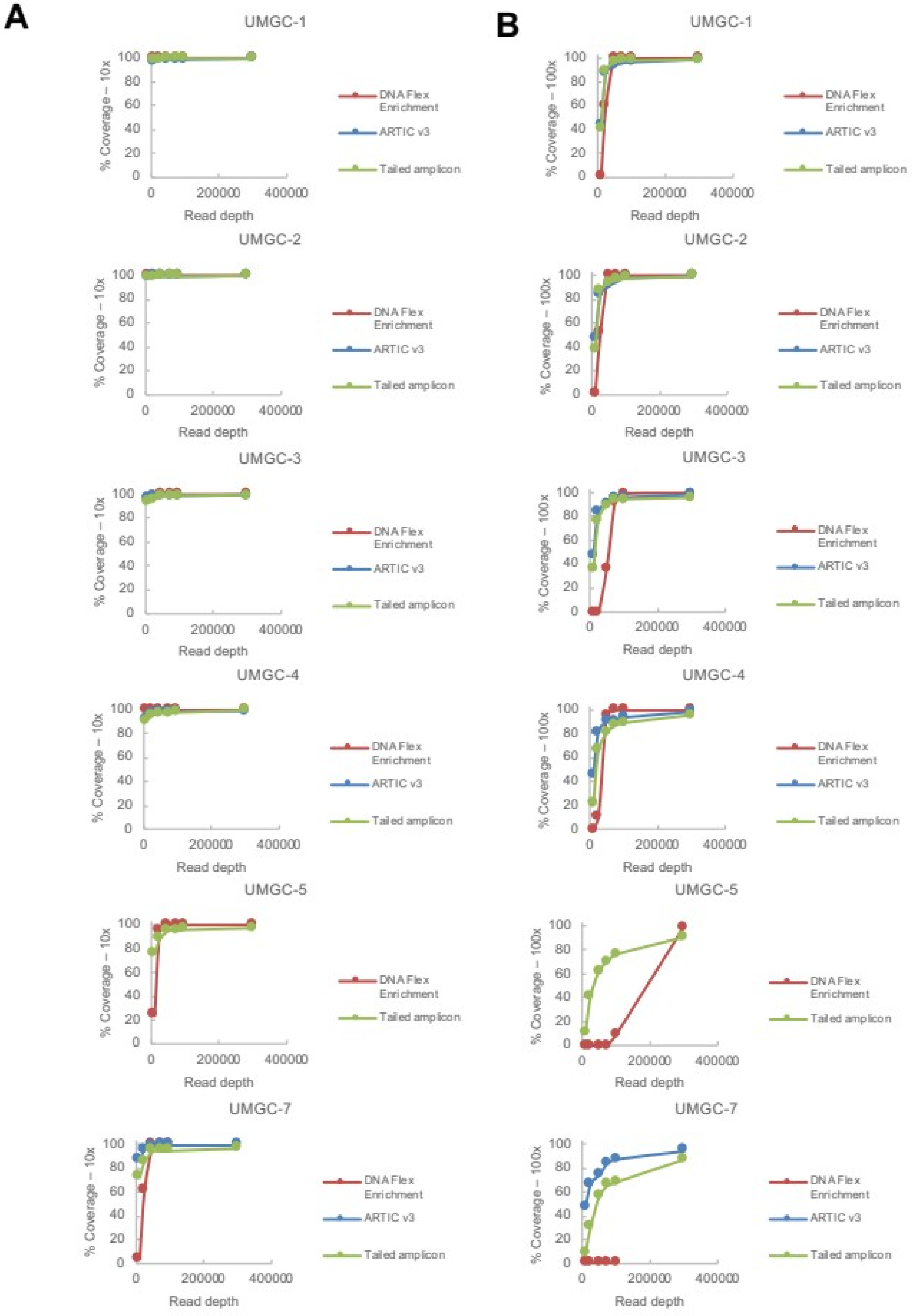

Supplemental Figure S3

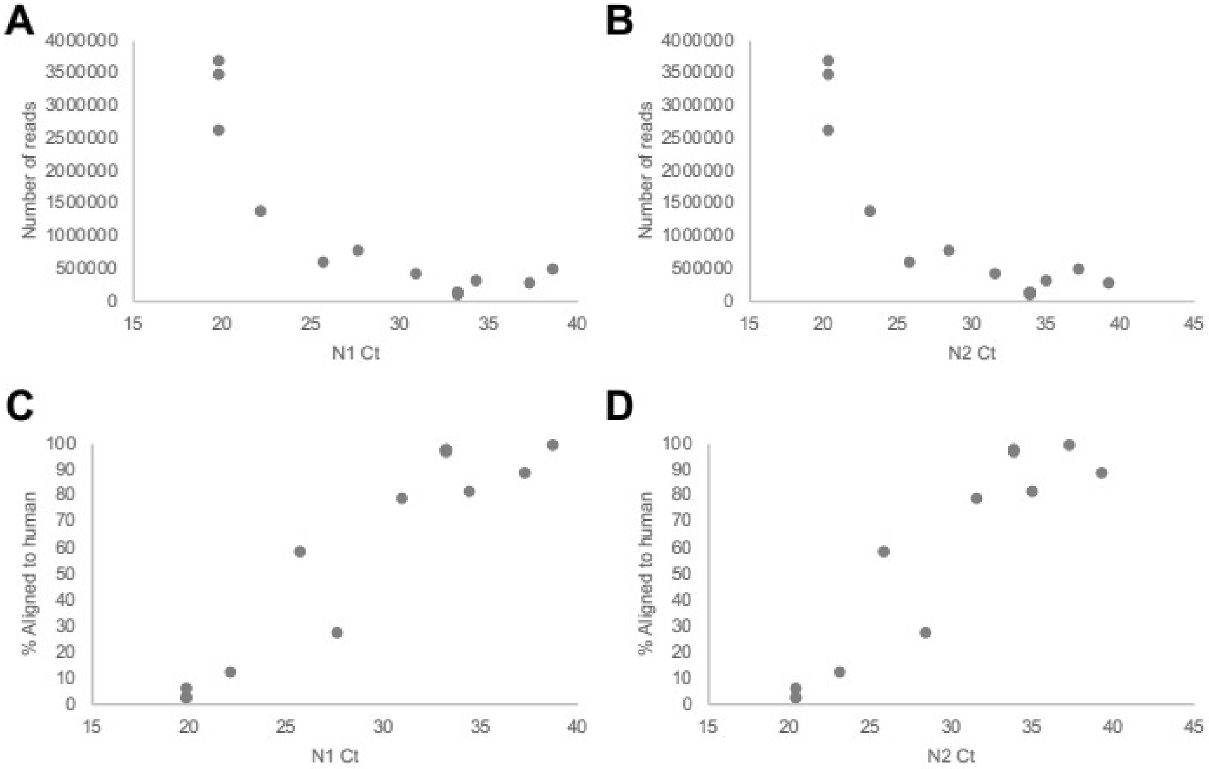

Supplemental Figure S4

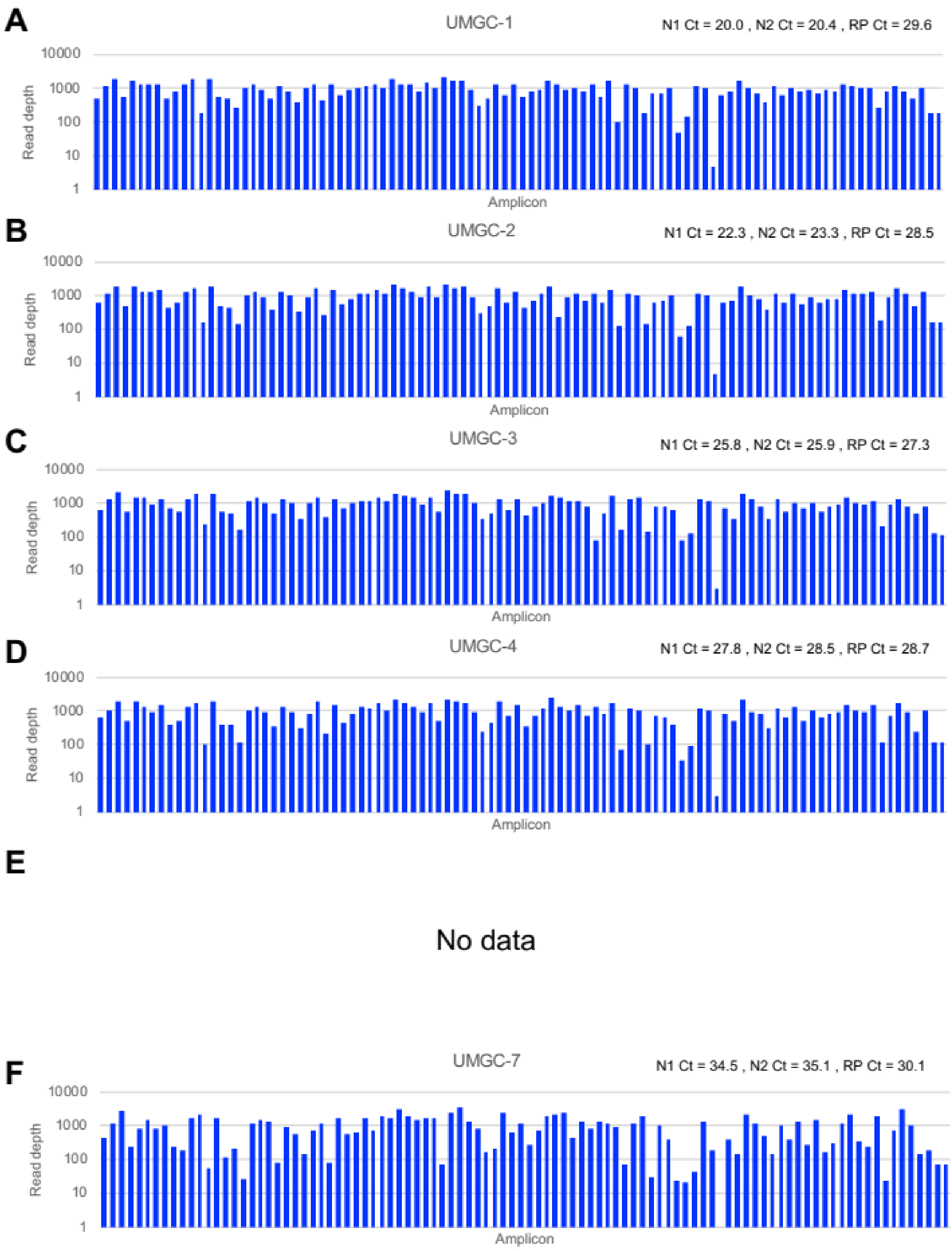

Supplemental Figure S5

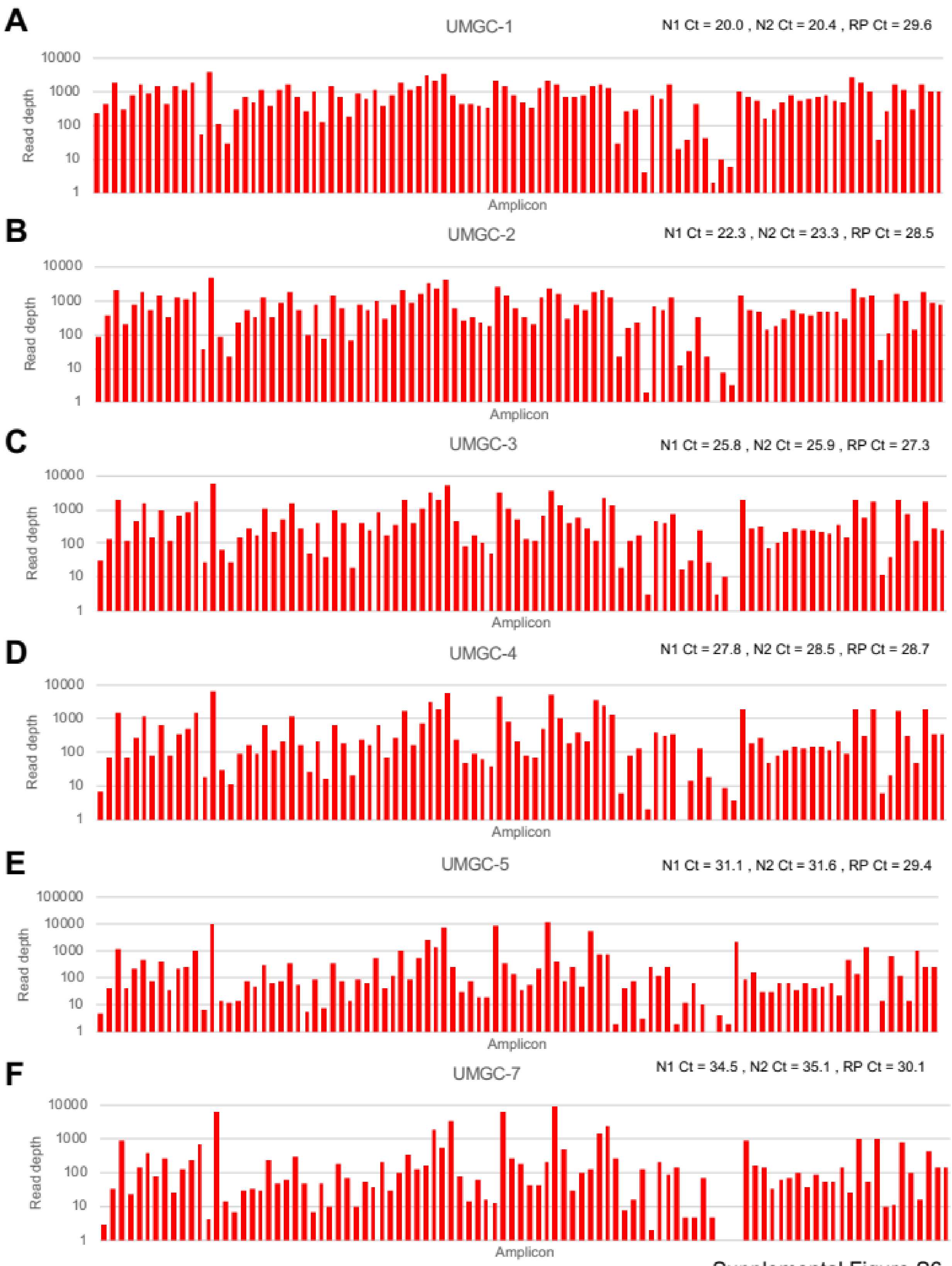

Supplemental Figure S6

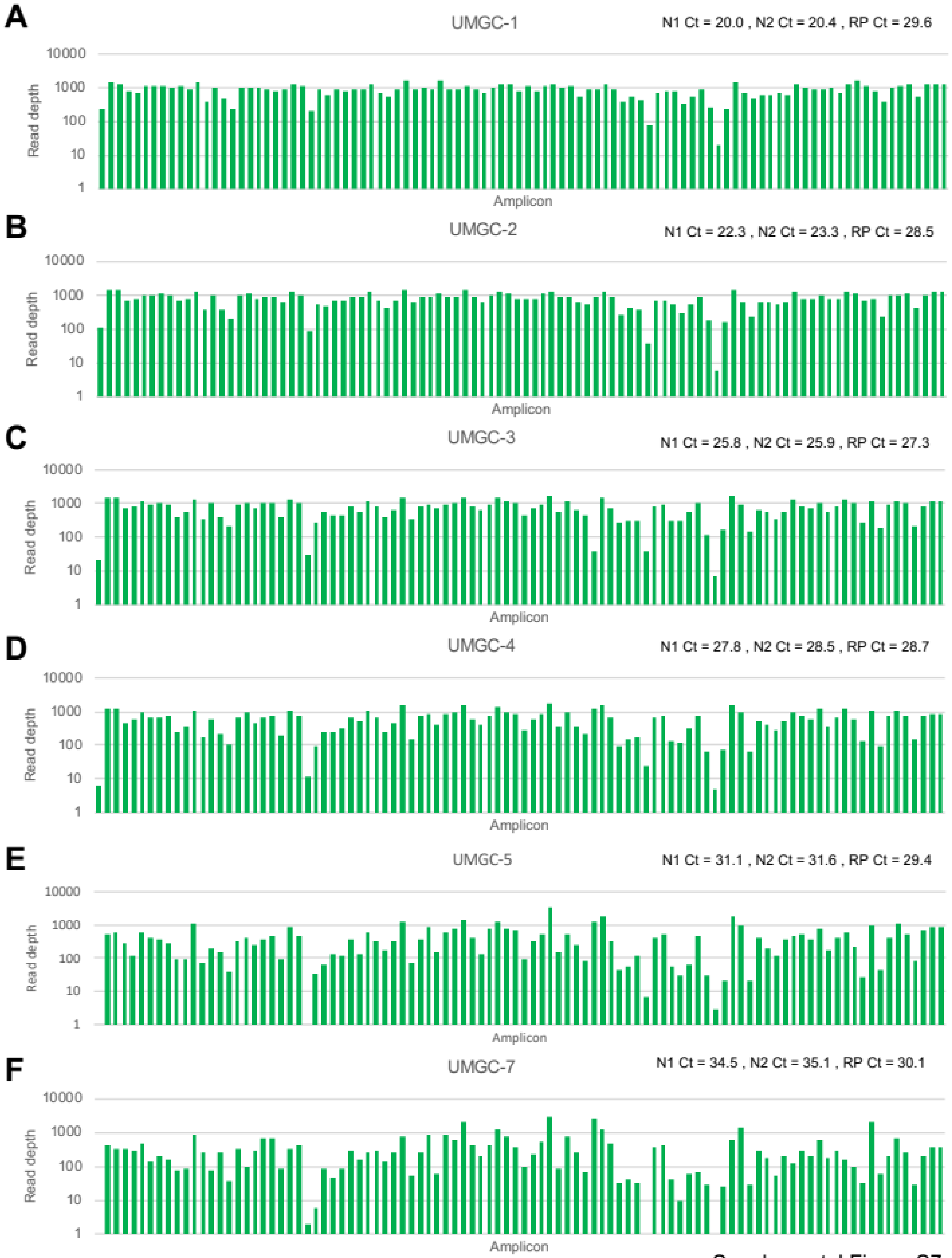

Supplemental Figure S7

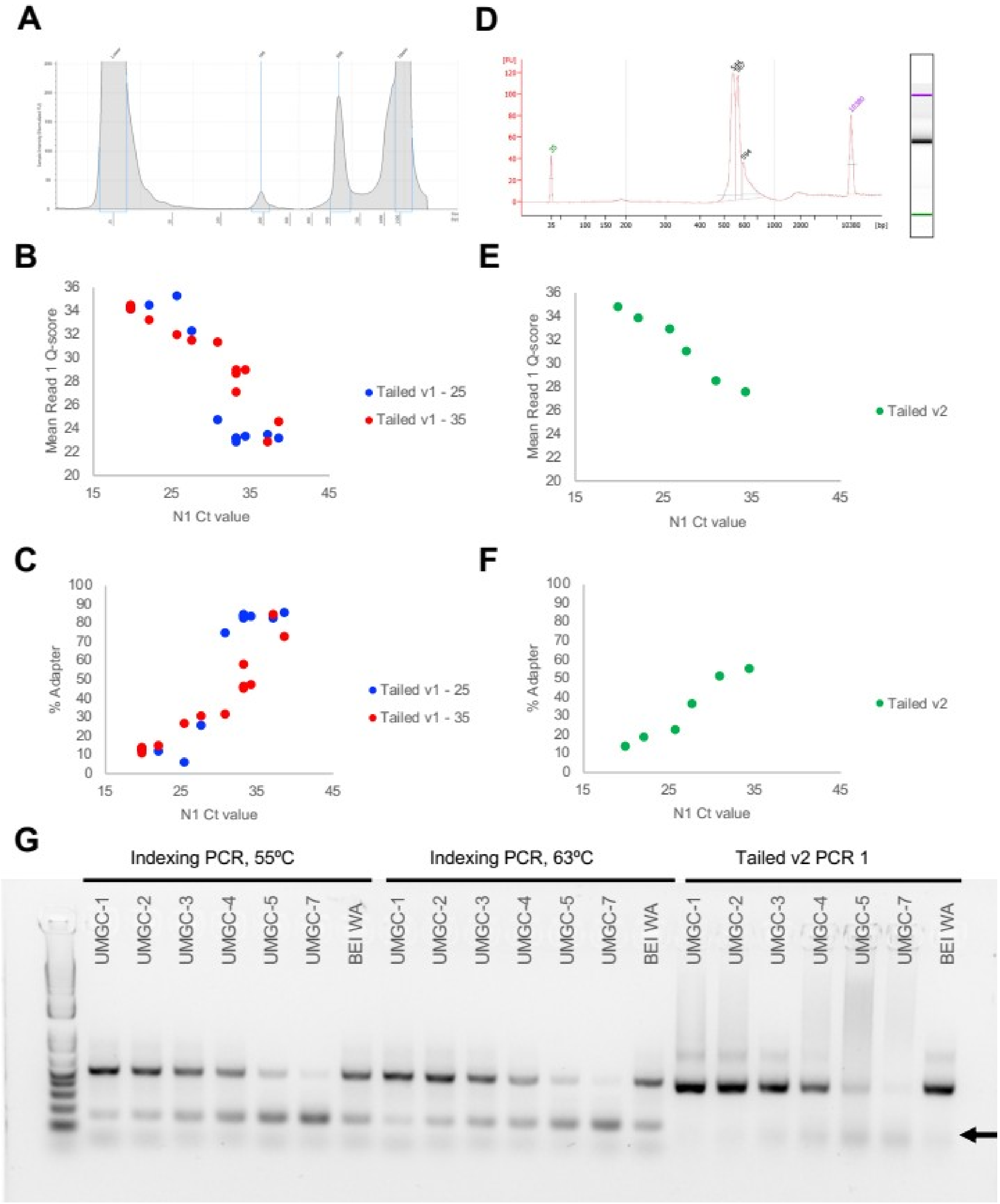

Supplemental Figure S8

BEI WA1 Isolate	Read depth					
**Method**	**10,000**	**25,000**	**50,000**	**75,000**	**100,000**	**300,000**
DNA Flex Enrichment	99.68	99.80	99.91	99.92	99.96	99.99
ARTIC v3	96.98	98.87	98.87	98.87	99.60	99.60
Tailed amplicon v1	82.80	90.16	92.33	95.58	97.09	98.67
Tailed amplicon v2	95.34	98.87	99.39	99.60	99.60	99.60

UMGC-1.1	Read depth					
**Method**	**10,000**	**25,000**	**50,000**	**75,000**	**100,000**	**300,000**
DNA Flex Enrichment	99.79	99.83	99.92	99.96	99.96	99.98
ARTIC v3	96.98	98.87	98.87	98.87	98.87	99.60
Tailed amplicon v1	89.05	91.44	95.97	96.16	97.09	No Data
Tailed amplicon v2	97.98	98.87	99.11	99.60	99.60	99.60

UMGC-1.2	Read depth					
**Method**	**10,000**	**25,000**	**50,000**	**75,000**	**100,000**	**300,000**
DNA Flex Enrichment	99.82	99.87	99.94	99.96	99.96	99.99
ARTIC v3	97.55	98.46	98.87	98.87	99.60	99.60
Tailed amplicon v1	88.32	92.98	97.09	97.09	97.09	98.87
Tailed amplicon v2	No Data	No Data	No Data	No Data	No Data	No Data

UMGC-1.3	Read depth					
**Method**	**10,000**	**25,000**	**50,000**	**75,000**	**100,000**	**300,000**
DNA Flex Enrichment	99.78	99.90	99.92	99.97	99.97	99.98
ARTIC v3	97.55	98.87	98.87	98.87	98.87	99.60
Tailed amplicon v1	88.43	91.75	95.97	96.44	97.09	98.87
Tailed amplicon v2	No Data	No Data	No Data	No Data	No Data	No Data

UMGC-2	Read depth					
**Method**	**10,000**	**25,000**	**50,000**	**75,000**	**100,000**	**300,000**
DNA Flex Enrichment	99.79	99.82	99.91	99.95	99.96	99.99
ARTIC v3	97.55	98.87	98.87	98.87	98.87	99.60
Tailed amplicon v1	85.67	90.55	95.16	95.30	96.10	97.97
Tailed amplicon v2	97.77	97.98	98.87	98.87	98.87	99.60

UMGC-3	Read depth					
**Method**	**10,000**	**25,000**	**50,000**	**75,000**	**100,000**	**300,000**
DNA Flex Enrichment	95.20	99.67	99.72	99.76	99.78	99.85
ARTIC v3	96.92	98.87	98.87	98.87	98.87	99.60
Tailed amplicon v1	77.42	86.93	91.19	95.19	97.08	97.09
Tailed amplicon v2	93.62	96.03	98.87	98.87	98.87	99.60

UMGC-4	Read depth					
**Method**	**10,000**	**25,000**	**50,000**	**75,000**	**100,000**	**300,000**
DNA Flex Enrichment	99.59	99.83	99.88	99.91	99.92	99.98
ARTIC v3	92.75	97.55	98.87	98.87	98.87	99.07
Tailed amplicon v1	70.19	82.33	87.95	90.53	92.22	97.17
Tailed amplicon v2	91.35	95.64	97.04	97.04	97.90	99.60

UMGC-5	Read depth					
**Method**	**10,000**	**25,000**	**50,000**	**75,000**	**100,000**	**300,000**
DNA Flex Enrichment	23.80	94.40	99.64	99.73	99.76	99.83
ARTIC v3	No Data	No Data	No Data	No Data	No Data	No Data
Tailed amplicon v1	47.73	71.14	79.60	85.90	89.59	94.30
Tailed amplicon v2	75.51	87.71	94.72	94.72	95.64	97.04

UMGC-7	Read depth					
**Method**	**10,000**	**25,000**	**50,000**	**75,000**	**100,000**	**300,000**
DNA Flex Enrichment	3.71	61.57	98.74	99.77	99.80	No Data
ARTIC v3	86.35	94.85	97.45	98.87	98.87	98.87
Tailed amplicon v1	44.61	67.23	78.64	81.52	87.27	94.23
Tailed amplicon v2	71.83	85.34	93.82	94.01	94.34	96.16

Supplemental Table 1

BEI WA1 Isolate	Read depth					
Method	10,000	25,000	50,000	75,000	100,000	300,000
DNA Flex Enrichment	0.34	47.86	98.47	99.65	99.69	99.90
ARTIC v3	52.00	88.20	92.74	96.98	97.31	98.87
Tailed amplicon v1	27.04	51.36	70.62	79.24	81.31	91.37
Tailed amplicon v2	26.07	77.56	92.72	94.30	95.64	97.90

UMGC-1.1	Read depth					
Method	10,000	25,000	50,000	75,000	100,000	300,000
DNA Flex Enrichment	0.98	59.71	99.67	99.76	99.77	99.87
ARTIC v3	43.49	87.11	93.73	95.99	96.56	98.87
Tailed amplicon v1	39.07	70.95	85.21	87.32	89.56	No Data
Tailed amplicon v2	40.70	89.30	96.07	97.98	97.98	98.87

UMGC-1.2	Read depth					
Method	10,000	25,000	50,000	75,000	100,000	300,000
DNA Flex Enrichment	0.17	67.20	99.67	99.76	99.79	99.87
ARTIC v3	50.01	88.12	93.73	95.99	97.34	98.87
Tailed amplicon v1	40.77	69.84	86.06	88.32	89.56	94.12
Tailed amplicon v2	No Data	No Data	No Data	No Data	No Data	No Data

UMGC-1.3	Read depth					
Method	10,000	25,000	50,000	75,000	100,000	300,000
DNA Flex Enrichment	0.18	66.77	99.45	99.74	99.79	99.88
ARTIC v3	45.93	87.25	93.73	96.12	97.55	98.87
Tailed amplicon v1	38.28	70.69	85.21	87.94	89.56	94.06
Tailed amplicon v2	No Data	No Data	No Data	No Data	No Data	No Data

UMGC-2	Read depth					
Method	10,000	25,000	50,000	75,000	100,000	300,000
DNA Flex Enrichment	0.11	51.99	99.57	99.72	99.76	99.88
ARTIC v3	46.38	83.56	90.90	95.14	97.55	98.87
Tailed amplicon v1	36.00	60.66	77.51	82.04	84.66	91.02
Tailed amplicon v2	37.17	86.44	93.68	95.64	97.12	98.87

UMGC-3	Read depth					
Method	10,000	25,000	50,000	75,000	100,000	300,000
DNA Flex Enrichment	0.00	0.55	36.81	92.97	98.96	99.69
ARTIC v3	47.74	84.52	91.08	95.48	95.99	98.35
Tailed amplicon v1	23.87	45.28	62.85	71.41	78.52	87.71
Tailed amplicon v2	35.91	77.21	90.22	93.62	94.66	96.06

UMGC-4	Read depth					
Method	10,000	25,000	50,000	75,000	100,000	300,000
DNA Flex Enrichment	0.11	10.69	95.99	99.59	99.68	99.83
ARTIC v3	45.73	81.81	89.94	91.44	93.69	98.35
Tailed amplicon v1	21.10	31.41	51.24	60.42	66.42	83.62
Tailed amplicon v2	21.70	66.16	80.76	86.95	89.11	95.64

UMGC-5	Read depth					
Method	10,000	25,000	50,000	75,000	100,000	300,000
DNA Flex Enrichment	0.12	0.12	0.12	0.22	8.88	99.36
ARTIC v3	No Data	No Data	No Data	No Data	No Data	No Data
Tailed amplicon v1	13.00	23.78	37.55	41.10	45.71	72.28
Tailed amplicon v2	10.90	41.17	62.87	69.83	76.16	90.67

UMGC-7	Read depth					
Method	10,000	25,000	50,000	75,000	100,000	300,000
DNA Flex Enrichment	0.12	0.12	0.12	0.12	0.61	No Data
ARTIC v3	47.09	66.34	74.03	83.90	87.29	93.98
Tailed amplicon v1	8.31	17.50	28.81	37.80	45.76	68.56
Tailed amplicon v2	8.73	30.59	56.22	65.21	67.99	86.97

Supplemental Table 2

25 PCR cycles
Tailed amplicon v1

	Read depth					
**Sample**	**10,000**	**25,000**	**50,000**	**75,000**	**100,000**	**300,000**
BEI WA1	89.23	96.08	98.03	98.87	98.87	99.60
UMGC-1.1	88.16	91.79	97.18	97.18	98.87	98.87
UMGC-1.2	87.71	95.23	97.97	98.87	98.87	98.87
UMGC-1.3	87.17	94.82	97.98	97.98	98.87	98.87
UMGC-2	89.20	94.37	97.94	98.87	98.87	98.95
UMGC-3	86.29	93.43	97.97	98.87	98.87	98.87
UMGC-4	85.85	92.18	96.33	98.81	98.87	No Data
UMGC-5	37.55	60.72	81.89	88.57	No Data	No Data
UMGC-7	5.74	21.75	37.67	No Data	No Data	No Data

Supplemental Table 3

25 PCR cycles
Tailed amplicon v1

	Read depth					
**Sample**	**10,000**	**25,000**	**50,000**	**75,000**	**100,000**	**300,000**
BEI WA1	30.54	53.25	79.17	85.55	89.40	97.97
UMGC-1.1	30.80	58.61	78.75	87.44	88.16	94.37
UMGC-1.2	29.79	54.69	77.77	86.47	88.16	94.37
UMGC-1.3	31.55	54.92	75.75	86.47	88.16	94.37
UMGC-2	25.93	48.02	71.93	86.37	87.32	94.37
UMGC-3	28.28	51.21	75.54	84.66	86.34	95.30
UMGC-4	23.98	46.50	65.27	79.29	86.60	No Data
UMGC-5	1.26	8.66	20.96	31.92	No Data	No Data
UMGC-7	0.00	0.00	1.62	No Data	No Data	No Data

Supplemental Table 4
